# Active Biodegradable Films Based on Sweet Lime Peel Residue and Its Effect on Quality of Fish Fillets

**DOI:** 10.3390/polym13081240

**Published:** 2021-04-12

**Authors:** Yasir Arafat, Ammar Altemimi, Anubhav Pratap-Singh, Laxmikant S. Badwaik

**Affiliations:** 1 Department of Food Engineering and Technology, School of Engineering, Tezpur University, Napaam, Assam 784028, India; yasir.arafatt@gmail.com; 2 Department of Food Science, College of Agriculture, University of Basrah, Basrah 61004, Iraq; ammar.ramddan@uobasrah.edu.iq; 3 Food, Nutrition & Health Program, Faculty of Land and Food Systems, The University of British Columbia, Vancouver, BC V6T 1Z4, Canada

**Keywords:** sweet lime peel, microwave extraction, active film, antimicrobial properties, shelf life

## Abstract

Residual sweet lime peels after the extraction of essential oil by solvent free microwave extraction were used for developing biodegradable film. Glycerol as a plasticizer, soya lecithin as an emulsifier and sweet lime essential oil (0, 1, 2 and 3%) as an active agent was employed. Developed films were analyzed for their mechanical, barrier and antimicrobial properties. The films (with 3% essential oil) which reported highest antimicrobial property against E. coli (24.24 ± 2.69 mm) were wrapped on fish fillet and stored at the refrigerated condition for 12 days. The quality of fish fillets was evaluated every 4 days and compared with polyethylene wrapped and control fish fillets. The active film wrapped sample showed less surface microbial count (3.28 ± 0.16 log cfu/cm^2^) compared to polyethylene wrapped sample. The hardness values were increased during storage and cohesiveness and springiness of all wrapped samples decreased from day 0 to day 12.

## 1. Introduction

Citrus juices are one of the most common fruit juices consumed globally, which also constitute a large amount of waste that largely goes to land-fills. Sweet lime (Citrus limetta) is most commonly known as “mousambi” in India. Sweet lime peel is a waste material generated from its processing industries and it is a good source of flavonoids, pectin and essential oil. Such citrus wastes are most popularly used for extraction of essential oil due to their antimicrobial, anxiolytic, antioxidant and anti-inflammatory activities. The oil extracted from citrus peel has a strong and desirable aroma with refreshing effect. There are many processes used for extraction of essential oil for citrus wastes such as solvent extraction, hydro-distillation, steam distillation, microwave extraction, etc. However, wastes/residues left with these processes are considered as secondary waste with no commercial value. Many researchers utilized citrus industry waste for extraction of essential oil [1,2,3,4,5]. However, they have not suggested the effective utilization of such secondary waste generated by these processes. One of the possible uses of such residues is development of biodegradable packaging.

Biopolymer materials are gaining large applications and slowly replacing the use of synthetic plastics due to its biodegradable nature. In manufacturing of biopolymer films size reduction of biopolymer particles is the key factor for its uniform distribution in film. Biopolymer films and coatings have shown great potential for their commercial application in food products but their utilization is not much popular in food industry [6]. High cost of biopolymer materials is the major drawback in practical application. Agricultural byproducts may be a feasible source for developing edible films and coatings with minimum cost of manufacturing. Various authors utilized fruit industry waste for developing biodegradable film [7,8,9,10,11]. In recent literatures, muskmelon seeds, pumpkin seeds and peels, blueberry pomace, shallot stalk waste, tamarind seed and seed mucilages have also been utlized for biodegradable film formation [12,13,14].

Essential oils derived from plant source have been widely used as antimicrobial and flavoring agents due to the presence of variable terpenoid and phenolic contents, and as a result, the anti-bacterial and anti-fungal activities of several essential oils have been well-studied. These have been successfully applied for organic preservation, when conventional processing solutions cannot be applied [15]. Antioxidants in such essential plant extracts are also known to slow down lipid oxidation and other deteriorative chemical reactions [16,17]. Essential oil of Artemisia fragrans were incorporated in chitosan coating for application on fresh chicken meat [18]. Clove essential oil in Alginate/κ-Carrageenan-based edible films and orange essential oil in carrageenan edible films have been incorporated for enhancing antioxidant–antimicrobial activity [19].

The application of edible or biodegradable films and coatings for a wide range of food products such as fresh, minimally processed vegetables, fruits, meat, and fish are gaining more appreciation, as it improves shelf-life of the product; they may also improve the texture and sensory characteristics. Edible films, coatings and biodegradable packaging produced from biological materials possess numerous advantages over other conventionally used synthetic packaging materials. The major function of these edible films and biodegradable packaging are to provide internal moisture or solute resistance with heterogeneous foods, individual protection of food pieces, encapsulation of functional food additives, etc.

In current study, residues left after extraction of essential oil from sweet lime peel by vacuum assisted solvent free microwave extraction method were utilized for developing biodegradable film. Additionally, the effect of essential oil incorporation on film properties and wrapping of fish fillet by the sweet lime peel based (SLP) film on the quality of the fish fillet during storage were studied.

## 2. Materials and Methods

### 2.1. Raw Materials

Sweet lime peel was collected from a juice vendor, Tezpur University, Assam, India and used for extraction of essential oils using vacuum-assisted solvent free microwave extraction in our earlier study [20]. The extraction conditions taken from an earlier study were 800 W microwave output power and 30 min extraction time. The residual sweet lime peels after extraction of essential oils were dried using a tray dryer at 50 °C for 48 h. The dried materials were ground to fine particles (<500 µm) using laboratory grinder and used for development of film. The essential oil extracted using this process was used as an active agent for film. Major chemicals required in this research, namely nutrient broth, nutrient agar, plate count agar, peptone, tetracycline, soya lecithin, glycerol and calcium chloride were supplied by Hi Media Laboratories, Mumbai, (India).

### 2.2. Development of Sweet Lime Peel Residue-Based Active Film

**Figure 1:**
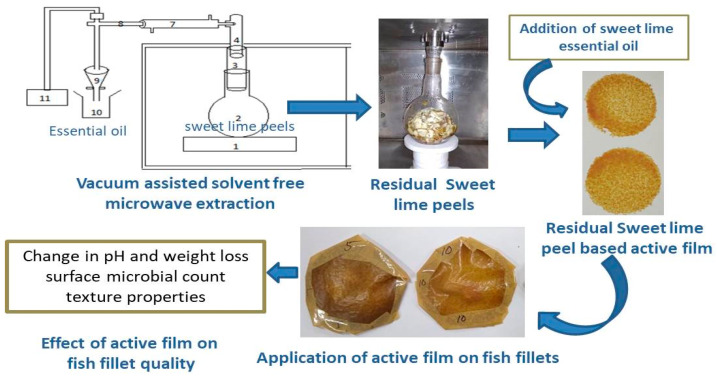
Development of sweet lime peel residue-based active film and its application on fish fillet.

The film forming solution was prepared by mixing 5 g of sweet lime peel residue powder in 100 mL of distilled water by using magnetic stirrer at 800 rpm for 15 min. All the solutions were heated at 85 °C for 45 min. Glycerol as a plasticizer (4 mL/100 mL of solution) and soya lecithin (2%) as an emulsifier were added in the suspension. The suspension was then cooled and sweet lime essential oil (0–3% of film forming material) as an active agent was added in it. These films were referred to as Film A, B, C and D for the film added with 0, 1, 2 and 3% essential oil, respectively. Further, the suspension was transferred into glass petri plates (15 mm dia.) and dried at 50 °C in a tray dryer. The dried films were removed from petri plates and immersed in 2% *w*/*w* CaCl_2_ solution for 10 min and dried again at 48 °C for 12 h. The dried films were kept for conditioning in desiccator for 72 h at 55 ± 1.2% RH and 20 ± 1 °C, and used for further testing [9]. The process of film development is shown in Figure 1.

### 2.3. Analysis of Sweet Lime Peel Residue-Based Active Film

The film developed using residue of sweet lime peel added with sweet lime essential oil at different concentration (0–3% of film forming material) were analyzed for their thickness, water vapor permeability (WVP), moisture absorption, solubility in water, textural properties, light transmittance and antimicrobial property (zone of inhibition).

#### 2.3.1. Film Thickness and Water Vapor Permeability

Film thickness was measured with a handheld micro meter (Alton M820-25, Shanghai, China) having a sensitivity of 0.01 mm. The water vapor permeability of films was evaluated using the method suggested by Jouki et al. [21]. A glass cup half-filled with anhydrous CaSO_4_ (0% RH) was covered with films and placed in a desiccator containing saturated K_2_SO_4_ solution (97% RH). The amount of weight absorbed by the cup was recorded as a function of time and the same was plotted. The water vapor transmission rate (WVTR) was calculated and used for determining the water vapor permeability (WVP) of film. WVP (g m^−1^ h^−1^ Pa^−1^) was calculated using the Equation (1).

(1) $$\mathrm{WVP}\;=\;\;\frac{\mathrm{WVTR}\;\times \;X}{P\;\left(R₁-\;R₂\;\right)}$$

where P is the saturation vapor pressure of water (Pa) at the test temperature (25 °C), R_1_ is the RH in the desiccator, R_2_ is the RH in the cup and X is the film thickness (m). Under these conditions, the driving force [P(R_1_ − R_2_)] is 3073.93 Pa.

#### 2.3.2. Moisture Absorption and Solubility of Film

The amount of moisture absorbed by film from surrounding controlled atmosphere (having relative humidity of 55% at 20 °C to 25 °C) were recorded and the moisture absorption capacity of film was calculated [22]. The dried films of 20 mm × 20 mm were first conditioned at 0% RH (CaSO_4_) for 24 h. The weight (W_o_) of films were taken and they were conditioned in a desiccator containing CaNO_3_ saturated solution at 20 °C to 25 °C to ensure a relative humidity of 55%. Each film was weighed at desired intervals (W_t_) until the equilibrium state was reached. The moisture absorption of the samples was calculated using Equation (2).

(2) $$\mathrm{Moisture}\;\mathrm{Absorption}\;(\%)\;=\;\frac{\mathrm{Wt}\;-\;\mathrm{Wo}}{\mathrm{Wo}}\;\times \;100$$

where W_t_ and W_o_ are the weights of the sample after t time at 55% RH and the initial weight of the sample, respectively. Where, W_t_ and W_o_ are the weights of the sample after t time at 55% RH and the initial weight of the sample, respectively.

The percentage of dry matter of film which was solubilized after 24 h immersion in water was used to calculate the solubility of film [23]. Film specimens were kept in a desiccator containing dry calcium sulphate (CaSO_4_) until they reached constant weight. Afterwards, about 500 mg of each films were immersed in beakers containing 50 mL of distilled water at room temperature for 24 h with periodical gentle manual agitation. The films were removed from the water and were placed back in the desiccator until they reached a constant weight to obtain the final dry weight of the film. The percentage of the total soluble matter (%TSM) of the films was calculated using Equation (3).

(3) $$\mathrm{TSM}\;(\%)\;=\;\;[\frac{\mathrm{Initial}\;\mathrm{dry}\;\mathrm{weight}\;-\;\mathrm{Final}\;\mathrm{dry}\;\mathrm{weight}}{\mathrm{Initial}\;\mathrm{Dry}\;\mathrm{weight}}]\;\times \;100$$

#### 2.3.3. Textural Properties and Transparency of Film

Texture Analyzer (TA-HDPlus, Stable Microsystems, Godalming, UK) with Kieffer Dough and Gluten Extensibility Rig (A/KIE) was used for determining tensile strength (kPa) and elongation at break (EB) of films. Tensile strength (kPa) was calculated by dividing the maximum force at break by the length and thickness of the film. Elongation at break was determined as percentage of change in length of film [12]. The transparency of film was calculated by measuring the light barrier properties of the film samples (2 × 40 mm) using UV-VIS spectrophotometer (Spectronic 20D+, Thermo Scientific, Waltham, MA, USA) at wavelengths of 560 nm [14]. The transparency of film was calculated using Equation (4).

(4) $$\;\;Transparency=\;\frac{Abs^{y}}{X}\;\times 100$$

where *Abs^y^* is the value of absorbance at 560 nm and *X* is the film thickness.

#### 2.3.4. Antimicrobial Property of Film

An agar diffusion assay was used for assessing the antimicrobial property of film against *Escherichia coli* in nutrient agar medium [9]. For diffusion tests, 0.2 mL of 105 colony forming units (cfu)/mL of each bacterial culture was plated. The inoculum was spread evenly throughout the plate and then let dry for 5 min in a biosafety hood. The 6 mm diameter disc of film was deposited over the inoculated agar with the film’s shiny side down. The plates were incubated at 35 °C for 48 h. The inhibition radius around the film disc (colony-free perimeter) was measured with a digital caliper in triplicate after 48 h of incubation, respectively. The inhibition area was then calculated.

#### 2.3.5. Thermal Properties of Film

The onset temperature and peak temperature i.e., melting point (*T*_m_) of all the film were measured by differential scanning calorimetry (DSC 214 Polyma, Netzsch, Germany). A 10 mg film was taken and heated at temperature ranges between 30 and 160 °C at 10 °C/min under nitrogen atmosphere flows at 40 mL/min. The maximum endothermic peak of temperature has been considered as the melting point [24].

#### 2.3.6. Infrared Spectroscopy

The infrared spectroscopy for all the film was obtained with FTIR spectrophotometer (Spectrum 100, PerkinElmer, Waltham, MA, USA). The equipment was operated with a scanning range of 4000–450 cm^−1^ and spectrum of 100. The films were ground to a fine powder and then the sample (clear glassy disk) for FTIR analysis was prepared by mixing powdered sample with an IR grade KBr using FTIR hand operated press at around 12,000 psi.

### 2.4. Application of Active Film on Fish Fillets and Their Quality Evaluation

**Figure 2:**
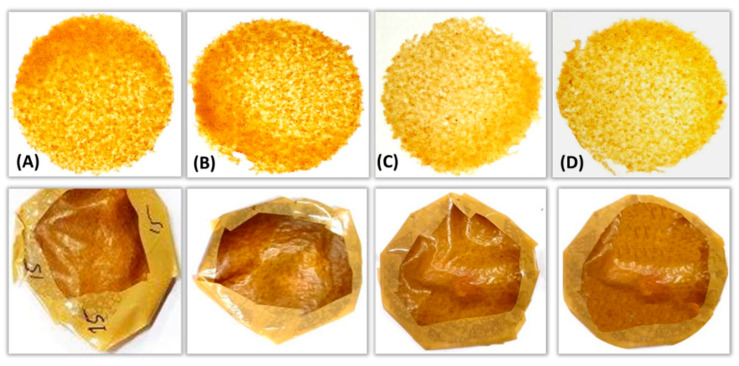
Images of sweet lime peel residue-based film (Film (**A**)—0% EO, Film (**B**)—1% EO, Film (**C**)—2% EO, Film (**D**)—3% EO) and packaged fish fillets.

Fresh fish (*Pangasius pangasius*) were procured from local fish market of Napaam, Assam, India. Fish fillet sample were taken for wrapping with different films (Figure 2) and stored at 4 °C for 12 days and at a 4-day interval, the quality of fish was studied (Figure 1). One set of fish fillets were wrapped with active film (having highest antimicrobial property), another set were wrapped with commercial polyethylene film and one set was kept without wrapping (control). The fish qualities were evaluated for its pH, texture, weight loss and surface microbial count. The control fish fillets were spoiled at 4 days of storage.

#### 2.4.1. pH and Weight Loss of Fish Fillets

Approximately 2 g fish fillet was mixed with 50 mL of distilled water. After 5 min mixing, the mixture was filtered, and then pH of the filtrate was measured using a calibrated digital pH meter (Mettler Toledo, Seven Easy S20, Gaithersburg, MD, USA) at room temperature [25]. Weight change of wrapped and control sample during 12 days of storage was measured. Weight loss was measured as the percentage weight lost from the original weight. Results of active film wrapped fish fillets were compared with polyethylene wrapped fish fillets and control fish fillets.

#### 2.4.2. Texture Profile Analysis of Fish Fillets

Texture profile analysis (TPA) was measured for each fish fillet. For TPA test, fish fillet was placed on the flat plate of a Texture Analyzer (TA-HDPlus, Stable Microsystems, Godalming, UK). This experiment included two consecutive compressions (40%) of the sample using a cylindrical probe (36 mm in diameter). The cylindrical probe was connected to a 5 kN load-cell with distortion rate of 0.2 mm/sec. The probe was forced into the sample and a shearing force acted to deform the sample. This shearing force generates a curve and consequently exhibits the distortion. The TPA parameters viz. hardness, cohesiveness and springiness were recorded.

#### 2.4.3. Surface Microbial Count

Surface microbial count of the control, wrapped with active film and wrapped with polyethylene fish fillet were observed for a period of 12 days at 4 day intervals. Wrapping from fish fillets were removed at the time of analysis. All wrapped and control fish fillet surface skin (2 cm^2^) was immersed in 90 mL sterile peptone water and vortexed for 2 min in a vortex shaker. Precisely 100 mL of this peptone water was taken and spread on plate count agar media and incubated for 36 h. Visible colonies were counted and logcfu/cm^2^ was calculated [9].

### 2.5. Statistical Analysis

All the analyses were performed in triplicates and data were reported as mean ± SD. Duncan’s multiple range tests were used to find the significant differences at a significance level of 0.05 using social sciences SPSS 11.5 statistical package.

## 3. Results and Discussion

### 3.1. Properties of Sweet Lime Peel Residue-Based Film

The properties of films developed using residue of sweet lime peel added with sweet lime essential oil at different concentration (i.e., 0, 1, 2 and 3%; films were referred to as Film A, B, C and D, respectively) were evaluated as shown in Table 1. The thickness of each film was uniform and found in the range of 0.34 to 0.35 mm. The water vapor permeability values were reported in the range of 1.25 × 10^−5^ to 1.36 × 10^−5^ g/Pa h m. The WVP values were found to increase slightly with increase in essential oil concentration. However, the effect was not significant. The reported WVP values were higher compared to WVP values of biodegradable films developed using potato peel (8.09–9.47 × 10^−9^ g/Pa h m), sweet lime peel (6.13–6.89 × 10^−9^ g/Pa h m) and pumpkin peel (9.82 × 10^−6^ g/Pa h m) [9,13]. Whereas, the values are in line with WVP values of muskmelon seed meal-based films (1.20–1.75 × 10^−5^ g/Pa h m) [12]. There were no significant differences reported for moisture absorption and solubility value of film and it was found in the range of 12.67–13.38% and 40.48–41.32%, respectively. The solubility of all film was found to be in line with solubility of film developed using potato peel, sweet lime pomace, sweet lime peel and pumpkin peel [9,13], and relatively lower in comparison with muskmelon seed meal-based film [12].

Tensile strength and elongation capacity of the film under stress conditions could be used to represent the film resistance to elongation and the amount of load it can handle. Tensile strength and elongation capacity of films were found in the range of 318.56–337.44 kPa and 3.84–4.64%, respectively. However, there was no significant difference among these values. These values are lower compared to tensile strength (657 ± 3.0 kPa) and elongation capacity (6.31 ± 0.23%) of pumpkin peel-based film [13]. This may have been due to degradation of pectin available in sweet lime peel during microwave treatment. The Film C showed the highest transparency of 37.4 ± 0.85% (Table 1), however there was no significant difference among these values for other films. Sweet lime peel residues were directly used for film formation; hence the transparency of film was found less. Transparency properties correlate with the light transmittance properties of the film [26].

### 3.2. Antimicrobial Properties of Film

Antimicrobial packaging or film having antimicrobial properties could reduce food losses and increase the shelf-life of food products [27]. Active films were prepared by incorporating different concentrations of essential oil extracted from sweet lime peel and their antimicrobial property was measured. The antimicrobial property (zone of inhibition) of different films against *E. coli* is given in Table 1. Antimicrobial properties of films were directly proportional to the concentration of essential oils. The film (D) having 3% essential oil concentration showed the highest antimicrobial property with a zone of inhibition of 24.24 ± 2.69 mm. However, the control film (Film A) did not show any suggestion of inhibition. Essential oils extracted from plants and spices are rich sources of bioactive compounds (terpenoids, phenolic compounds, etc.) which have been recognized as antimicrobial agents [28]. The film with the highest antimicrobial property (Film D) was chosen for the wrapping of the fish fillet.

### 3.3. Differential Scanning Calorimetry

**Figure 3:**
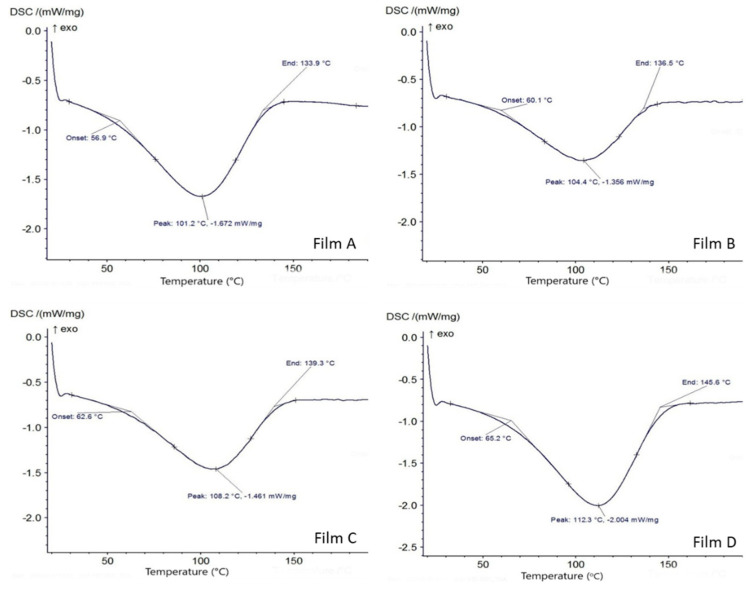
Differential Scanning Calorimetry (DSC) curve for sweet lime peel residue-based film added with 0% EO (Film **A**), 1% EO (Film **B**), 2% EO (Film **C**) and 3% EO (Film **D**).

The analysis of thermal attributes of the film is important in characterizing their application and processing temperature limit. The onset temperature and peak temperature i.e., melting point (*T*_m_) of different composition of the film were measured using DSC. The onset temperatures of a process for different films were in the range of 56.9 to 65.2 °C. The melting point (*T*_m_) is related to the maximum point (peak temperature) in the first endothermic peak and it was found in the range of 101.2 to 112.3 °C (Figure 3). The melting point of film was increased with increase in essential oil concentration and it was found highest in Film D.

### 3.4. Fourier Transform Infrared Spectroscopy of Film

**Figure 4:**
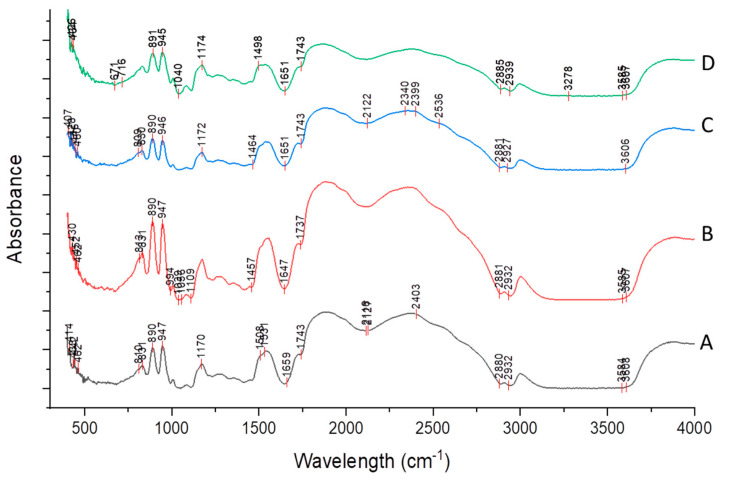
FTIR spectroscopy for sweet lime peel residue-based film added with 0% EO (Film **A**), 1% EO (Film **B**), 2% EO (Film **C**) and 3% EO (Film **D**).

FTIR spectroscopy is useful to investigate the chemical components and interactions by streaming infrared wave across the material. As seen from Figure 4, there are no significant changes in spectra observed with change in essential oil concentration in sweet lime peel residue-based film. The first broad absorbance peak is in the 3550–3200 cm^−1^ range, which can be related to O–H stretch and N–H stretch due to presence of glycerol and water. The absorbance around 2880 cm^−1^ may be due to C–H stretching. The peak at 1740 cm^−1^ related to C=O stretching, showing presence of aldehyde groups and the strong peak around 1650 cm^−1^ related to C=C stretching. The peaks at 1465 and 1450 cm^−1^ are due to C–H bending showing the presence of methyl group. The peaks at 1085–1050 cm^−1^ are due to the C–O stretching, and are characteristic bands of cellulose and hemicellulose from lignocellulosic materials.

### 3.5. Effect of Active Film on Fish Fillet Quality

The effect of wrapping of active film on fish fillets quality during their storage were studied and compared with fish fillets wrapped with polyethylene and control fish fillets.

#### 3.5.1. Change in pH and Weight Loss

The quality of fish is generally determined by its freshness and pH. The changes in pH of fish are related to the post-mortem evolution of the flesh. It is influenced by the species, diet, seasons, level of activity or stress [29]. Table 2 shows the change in pH and weight loss of wrapped and control fish fillet sample. The initial pH of the fresh fish fillet samples at day 0 was 6.21. The pH of active film wrapped and polyethylene wrapped fish fillets showed almost same trends of changes. The pH of active film and polyethylene wrapped fish fillets decreased significantly (*p* ≤ 0.05) from day 0 to day 8, and slightly increased after day 8 until end of storage. Active film and polyethylene wrapped fish fillets showed the highest pH of 6.13 and 6.10, respectively, at the end of storage. A decrease in pH of fish fillet may be due to the glycolysis that occurs after death, which leads to the accumulation of lactic acid in muscle at early stage of storage [30]. This might be also due to the dissolution of CO_2_ in the aqueous phase of the muscle tissue, resulting in the formation of carbonic acid in the muscle tissue [23]. At the later postmortem stage, pH increased because of the decomposition of nitrogenous compounds (protein). This might lead to the formation of volatile base components, such as ammonia, dimethylamine and trimethylamine as a result of endogenous microbial and/or enzymatic activity [31].

The percentage of weight loss in fish fillets wrapped with active film and polyethylene found to be increased with storage time and maximum weight loss was 31.42 ± 1.12% and 22.52 ± 1.42%, respectively, at 12 days of storage. Wrapping of fish fillet with active film and polyethylene significantly (*p* ≤ 0.05) reduced the weight loss compared to control fish fillets; it may be attributed to better WVP of the films, which restricted the moisture loss from fish fillet. Polyethylene showed more moisture transfer resistance compared to active film. The findings are in line with study of Das et al. [11]. They reported weight loss of around 11% was reported in unwrapped fish sample and around 8% in case of wrapped fish sample at the end of 4th day of storage at room temperature.

#### 3.5.2. Change in Surface Microbial Count

Surface microbial count of fish fillet wrapped with active film, polyethylene and control fish fillet during 12 days of storage are given in Table 3. The microbial count increased significantly (*p* ≤ 0.05) in the control and polyethylene wrapped fish fillet sample during 12 days of storage from 4.35 to 7.83 (0 to 4 days of storage) and 14.22 log cfu/cm^2^, respectively. However, fish fillet wrapped in active film samples showed a significant decrease (*p* ≤ 0.05) in surface microbial load from 4.35 to 3.28 log cfu/cm^2^. This might be due to the addition of the antimicrobial agent in the film forming solution, as this antimicrobial agent proved effective against *E. coli* earlier. This film was capable of reducing the microbial growth and kept the surface micro flora in control. Remya et al. [32] utilized chitosan-based film containing ginger essential oil as an active antimicrobial agent for preventing surface spoilage of fish. They also found that the film has good inhibition against LAB and *Brochothrix thermosphacta*.

#### 3.5.3. Change in Texture Properties

Texture properties of fish flesh depend on seasonality, protein and lipid content and quality. Texture properties of fish flesh are influenced by many intrinsic biological factors such as muscle fiber density, both fat and collagen content, as well as the post-mortem treatment, microbiological and autolytic processes caused by the death of the fish, which bring degradation of myofibrillar protein, following muscle softening [33].

The potential of films, used as wrapping material, to prevent the hardening of fish fillets during 12 days of storage was examined (Table 4). The hardness values were increased significantly (*p* ≤ 0.05) from 3.03 ± 0.52 to 9.64 ± 0.89% and 5.09 ± 0.34% for active film and polyethylene wrapped fish fillets, respectively. This can be correlated with the weight loss of fish fillets during storage [11]. In case of polyethylene wrapped sample, hardness was comparatively less as compared to active film sample. This may be due to lower water vapor permeability of the polyethylene. Additionally, after 8 days of storage, fish fillet wrapped with polyethylene showed a reduction in hardness. This reduction of hardness of fish fillet during storage might be due to the enzymatic degradation of muscle proteins [26]. The cohesiveness and springiness of all samples decreased from day 0 to day 12 (Table 4). This might be because of the change in the internal bonding and loss of elasticity of fish muscle. The reduction in textural values of fish fillet during storage might be affected by microbial or enzymatic activities [21]. All textural parameters of wrapped fish fillet showed lower reduction than the control fish fillet.

## 4. Conclusions

The residue left after the essential oil extraction from sweet lime peel was successfully used for developing active film incorporated with essential oil (0–3%). It was observed that the film that had the highest concentration of essential oil had the maximum antimicrobial property and other properties of film did not vary too much due to incorporation of essential oil in the film. Melting point of film was found to increase with an increase in essential oil concentration. The active film was found more resistant against micro-organism when fish fillets packed in it and stored for 12 days. Again, less change occurred in the textural properties of fish fillets during storage. Essential oil incorporated film showed an acceptable antimicrobial property which will have a promising future in food packaging. More research is required to attract consumers by developing new strategies on better appearance and other properties of the film.

## Figures and Tables

**Table 1:** Properties of sweet lime peel residue-based film added with sweet lime essential oil.

Film Name	Essential Oil Concentration (%)	Thickness (mm)	WVP (g/Pa h m)	Moisture Absorption (%)	Solubility (%)	Tensile Strength (kPa)	Elongation (%)	Transparency (%)	Zone of Inhibition (mm)
A	0	0.35 ± 0.019 ^a^	1.25 ± 0.11 (× 10^−5^) ^a^	12.67 ± 0.42 ^a^	41.32 ± 0.81 ^a^	337.44 ± 9.28 ^a^	4.64 ± 1.51 ^a^	32.4 ± 0.56 ^a^	0
B	1	0.34 ± 0.017 ^a^	1.30 ± 0.09 (× 10^−5^) ^a^	13.14 ± 0.10 ^a^	41.26 ± 1.03 ^a^	325.00 ± 8.44 ^a^	4.09 ± 2.03 ^a^	34.6 ± 0.47 ^a^	08.20 ± 1.41 ^a^
C	2	0.34 ± 0.014 ^a^	1.32 ± 0.13 (× 10^−5^) ^a^	12.89 ± 0.27 ^a^	40.48 ± 0.68 ^a^	318.56 ± 8.52 ^a^	3.84 ± 1.56 ^a^	37.4 ± 0.85 ^a^	18.12 ± 2.31 ^b^
D	3	0.35 ± 0.012 ^a^	1.36 ± 0.12 (× 10^−5^) ^a^	13.38 ± 0.67 ^a^	41.04 ± 0.036 ^a^	322.44 ± 10.52 ^a^	3.94 ± 1.57 ^a^	35.9 ± 0.64 ^a^	24.24 ± 2.69 ^c^

All data are the mean ± SD of three replicates. Mean followed by different letters in the same column differs significantly (*p* ≤ 0.05).

**Table 2:** Change in pH and weight loss of fish fillets qualities during storage.

Fish Fillets Quality	Storage Time (Days)	Control Fish Fillets	Fish Fillets Wrapped with Active Film	Fish Fillets Wrapped with Polyethylene
pH	0	6.21 ± 0.02 ^ap^	6.21 ± 0.02 ^ap^	6.21 ± 0.02 ^ap^
	4	6.14 ± 0.01 ^bp^	6.17 ± 0.03 ^bp^	6.15 ± 0.01 ^bp^
	8	NA	6.12 ± 0.01 ^cp^	6.07 ± 0.02 ^cr^
	12	NA	6.13 ± 0.01 ^cq^	6.10 ± 0.01 ^dp^
Weight Loss (%)	0	0	0	0
	4	29.46 ± 1.20 ^p^	21.64 ± 1.04 ^bq^	16.26 ± 0.74 ^br^
	8	NA	27.63 ± 1.09 ^cq^	19.86 ± 0.61 ^cr^
	12	NA	30.42 ± 1.12 ^dq^	22.52 ± 1.42 ^dr^

Values expressed are mean ± standard deviation. Means in the row with different superscript (^p^, ^q^, ^r^) and in column (^a^, ^b^, ^c^, ^d^) are significantly different at *p* ≤ 0.05 (this is calculated separately for each quality parameters), NA stands for Not Applicable as sample was spoiled after 4 days of storage.

**Table 3:** Change in surface microbial count of fish fillet quality during storage.

Fish Fillets Quality	Storage Time (Days)	Control Fish Fillets	Fish Fillets Wrapped with Active Film	Fish Fillets Wrapped with Polyethylene
Surface Microbial count (Logcfu/cm^2^)	0	04.35 ± 0.71 ^ap^	04.35 ± 0.71 ^ap^	04.35 ± 0.71 ^ap^
	4	07.83 ± 0.89 ^bp^	03.42 ± 0.34 ^bq^	05.69 ± 0.82 ^ar^
	8	NA	03.36 ± 0.24 ^bq^	09.73 ± 1.06 ^br^
	12	NA	03.28 ± 0.16 ^bq^	14.22 ± 1.42 ^cr^

Values expressed are mean ± standard deviation. Means in the row with different superscript (^p^, ^q^, ^r^) and in column (^a^, ^b^, ^c^) are significantly different at *p* ≤ 0.05. NA stands for Not Applicable as sample was spoiled after 4 days of storage.

**Table 4:** Change in texture properties of fish fillets qualities during storage.

Fish Fillets Quality	Storage Time (Days)	Control Fish Fillets	Fish Fillets Wrapped with Active Film	Fish Fillets Wrapped with Polyethylene
Hardness, N	0	3.03 ± 0.41 ^ap^	3.03 ± 0.41 ^ap^	3.03 ± 0.41 ^ap^
	4	7.45 ± 0.32 ^bp^	5.09 ± 0.17 ^bq^	4.36 ± 0.20 ^br^
	8	NA	8.24 ± 0.46 ^cq^	6.24 ± 0.12 ^cr^
	12	NA	9.64 ± 0.52 ^dq^	5.09 ± 0.29 ^dr^
Cohesiveness	0	0.43 ± 0.03 ^ap^	0.43 ± 0.03 ^ap^	0.43 ± 0.03 ^ap^
	4	0.38 ± 0.02 ^bp^	0.40 ± 0.03 ^bp^	0.41 ± 0.04 ^ap^
	8	NA	0.36 ± 0.01 ^bq^	0.38 ± 0.02 ^aq^
	12	NA	0.29 ± 0.02 ^cp^	0.33 ± 0.03 ^aq^
Springiness	0	0.87 ± 0.04 ^ap^	0.87 ± 0.04 ^ap^	0.87 ± 0.04 ^ap^
	4	0.81 ± 0.05 ^ap^	0.83 ± 0.03 ^ap^	0.82 ± 0.02 ^ap^
	8	NA	0.79 ± 0.05 ^ap^	0.77 ± 0.03 ^ap^
	12	NA	0.73 ± 0.02 ^ap^	0.70 ± 0.02 ^ap^

Values expressed are mean ± standard deviation. Means in the row with different superscript (^p^, ^q^, ^r^) and in column (^a^, ^b^, ^c^, ^d^) are significantly different at *p* ≤ 0.05 (this is calculated separately for each quality parameters), NA stands for Not Applicable as sample was spoiled after 4 days of storage.

## Data Availability

All data is included within the manuscript.
